# Interleukin-6 as a critical inflammatory marker for early diagnosis of surgical site infection after spine surgery

**DOI:** 10.1007/s15010-024-02271-4

**Published:** 2024-05-06

**Authors:** Paul Jonathan Roch, Carolin Ecker, Katharina Jäckle, Marc-Pascal Meier, Maximilian Reinhold, Friederike Sophie Klockner, Wolfgang Lehmann, Lukas Weiser

**Affiliations:** 1 Department of Trauma Surgery, Orthopaedics and Plastic Surgery, University of Göttingen, Robert-Koch-Str. 40, 37075 Göttingen, Germany; 2 Department of Surgery, University Medical Center Mannheim, Theodor-Kutzer-Ufer 1-3, 68167 Mannheim, Germany

**Keywords:** Surgical site infection, Spine surgery, Inflammatory marker, Interleukin-6

## Abstract

**Purpose:**

Early diagnosis of surgical site infections (SSIs) could prevent surgical revision. Inflammatory markers (IMs), such as procalcitonin (PCT), interleukin-6 (IL-6), and tumor necrosis factor α (TNF-α), seem more accurate in diagnosing SSI than C-reactive protein (CRP) and white blood cell (WBC) count. The aim was to compare the predictive values of CRP, WBC count, PCT, IL-6, and TNF-α in SSI detection.

**Methods:**

A total of 130 patients undergoing dorsal spondylodesis from 2019 to 2024 were enrolled in a prospective diagnostic study at a maximum care spine center. IMs were measured preoperatively and on the postoperative days (PODs) 1, 2, 3, 5, and 7. Patients with high suspicion of SSI underwent revision surgery. SSI was diagnosed when the microbiological evidence was positive. Patients were divided a posteriori into the non-infection and infection groups.

**Results:**

IMs of 118 patients (66.9 ± 13.0 years, 61.0% females) were measured. Fifteen of the 118 patients (12.7%) developed an SSI. The groups differed with respect to existing hypertension, number of instrumented segments, region of surgery, CRP_POD1,7_, PCT_POD7_, and IL-6_POD3,5,7_. Binary logistic regression for SSI detection including these parameters showed an area under the curve (AUC) of 0.88 (95% CI 0.79–0.98; *P* < 0.001). The main effect for SSI detection was maintained by IL-6_POD7_ (odds ratio = 1.13; 95% CI 1.05–1.23; *P* = 0*.*001), which itself showed an AUC of 0.86 (95% CI 0.75–0.97).

**Conclusion:**

Compared to CRP, WBC count, PCT, and TNF-α, IL-6 seems to be the critical IM for the early detection of an SSI.

**Trial registration:**

drks.de: DRKS00033773, date of registration: 29.02.2024, retrospectively registered; Postoperative Markers of Inflammation in Spine Surgery (POMIS) Trial.

## Introduction

Surgical site infection (SSI) is a serious complication of dorsal spondylodesis, with significant short- and long-term consequences for patients and considerable socioeconomic burden [1, 2]. Despite attempts to develop SSI risk stratification systems, the lack of conclusive parameters for revision or “watchful waiting” hampers clinical decision-making [2–5]. The gold standard for SSI diagnosis is still deep tissue biopsy and microbiologic culture, although this is an a posteriori tool. Therefore, routine clinical and laboratory monitoring remains the most common method of assessing postoperative infection. Imaging modalities such as magnetic resonance imaging (MRI) are associated with a high false-positive rate for the early diagnosis of SSI because they are confused with normal postoperative findings [2].

Standard inflammatory markers (IMs) for routine laboratory testing include white blood cell (WBC) count and C-reactive protein (CRP). CRP has been shown to be superior to WBC count in the assessment of SSI [6, 7]. Although specific postoperative CRP kinetics may indicate an SSI at approximately postoperative day (POD) 10 or later [8], there is no true reference test that allows for safe and early SSI diagnosis.

Other cytokines have been shown to aid in early SSI diagnosis. Interleukin-6 (IL-6), which is released by inflammatory cells, fibroblasts, and endothelial cells, stimulates the release of CRP and is elevated in the early stages of inflammation [9]. Rettig et al. [10] found an association between high IL-6 levels and postoperative complications in major abdominal surgery, which helps to differentiate between patients at low and high risk of complications. Tumor necrosis factor-alpha (TNF-α), a multifunctional cytokine produced by various cells of the immune system, plays a critical role in apoptosis, inflammation, and immunity [11]. There is evidence that TNF-α induces concentration-dependent CRP secretion from hepatocytes [9]. Dimopoulou et al. [12] observed that the balance between TNF-α and IL-10 appears to determine the occurrence of postoperative complications in major abdominal surgery. Procalcitonin (PCT) is produced by epithelial cells in response to bacterial inflammation [13]. Unlike CRP, PCT does not increase physiologically after surgery [14]. Aouifi et al. [15] showed that PCT is a reliable and more relevant marker of SSI after cardiac surgery than CRP.

Regarding spine surgery, information on the predictive value of IL-6, TNF-α, and PCT for early SSI diagnosis is lacking. The aim of this study was to investigate the comparative value of CRP, IL-6, TNF-α and PCT for the early detection of SSI after spinal surgery.

## Materials and methods

### Study design

This study was designed as a diagnostic prospective study, based on the Postoperative Markers of Inflammation in Spine Surgery (POMIS) Trial. It was approved by the institutional review board of the University of Göttingen (Ethics Committee Göttingen, approval number 31/8/19), and the trial was registered at drks.de (DRKS00033773). The study took place from September 2019 to January 2024. All the participants gave their written informed consent to participate in this study.

### Patients

A total of 130 patients were included in this study. The inclusion criteria were: (1) individuals aged 18 years and older; (2) had undergone open reduction and dorsal spondylodesis in their cervical, thoracic, or lumbar spine; and (3) the operation was conducted by one of three senior operators at a maximum care spine center (Level 1) of the German Spine Society (DWG®). The exclusion criteria were: (1) polytrauma patients (injury severity score > 16); (2) patients with open spine fractures; (3) spinal conditions affected by tumorous diseases (either primary tumors or metastases); (4) preexisting spine infections; (5) documented infections, inflammatory diseases, or elevated IMs before the surgery; and (6) incomplete patient records. 12 patients were excluded due to incomplete patient records.The indications for the procedures were degenerative pathologies (i.e., spondylolisthesis, scoliosis, osteochondrosis, and stenosis) and traumatic pathologies (fractures). Patient-related criteria, including age, sex, body mass index (BMI), American Society of Anesthesiologists (ASA) score, comorbidities, and surgical criteria, were systematically recorded. Additionally, information on the number of bridged segments and the operative region (i.e., cervical, thoracic, or lumbar) were documented.

One hour before each operation, preoperative intravenous antibiotics were administered, typically, 2 g of cefazolin, or 600 mg of clindamycin in case of penicillin allergy. Vancomycin powder was not applied topically on the wound. The drains were routinely removed on POD 2, and the wounds were inspected every two days postsurgery. Patients with high clinical suspicion of SSI (i.e., prolonged wound secretion) and common laboratory suspicion of SSI (i.e., increasing CRP values) underwent revision surgery. There was no antimicrobial administration up to the diagnosis of an SSI, except for patient 12, where antibiotic treatment was started at about postoperative day 28 by another hospital.

Patients were categorized into the infection group if a bacterium was detected during the revision surgery or if pus was seen during the operation. For the microbiological examinations, at least five aerobic and anaerobic tissue biospies were cultured (i.e., incubated for at least three weeks), and their histopathology was obtained according to the recommendations of the American Academy of Orthopaedic Surgeons [16].

### Inflammatory markers

Blood serum samples were collected either on the day before the operation or on the day of the operation, and on PODs 1, 2, 3, 5, and 7. The following IMs were determined: CRP, WBC count, PCT, TNF-α, and IL-6. The following devices and reference ranges were used for the laboratory analysis. CRP was measured via immunoturbidimetry (Abbott, Wiesbaden, Germany) with a reference range of < 5.0 mg/l. WBC count was determined using the CELL-DYN hematology analysis system (Abbott) with a reference range of 4.0–11.0 × 10^3^/µl. PCT was measured using Chemiluminescence Microparticle Immunoassay (Abbott) with a reference range of < 0.07 µg/l. TNF-α was determined using the IMMULITE 1000 Immunoassay System (Siemens, Munich, Germany) with a reference range of < 8.1 pg/ml. IL-6 was determined using the Electrochemiluminescent Immunoassay method (Abbott) with a reference range of < 7.0 pg/ml.

### Outcomes

The primary endpoint was the measurement of the IMs assessed preoperatively and at PODs 1, 2, 3, 5, and 7. The secondary endpoint was revision surgery due to the development of an early SSI within 30 PODs, as defined by the U.S. Centers for Disease Control and Prevention [17]. The a priori hypothesis was that PCT, IL-6, and TNF-α would have better predictive values than CRP and WBC count in early SSI diagnosis.

### Statistics

CRP was selected for sample size determination as it is the most common and routine IM. The sample size was determined using G-Power [18], assuming significantly different CRP values between the non-infection group and the infection group on POD 7 (66.5 ± 48.3 vs. 131.4 ± 79.7 mg/l) and POD 8 (55.4 ± 45.5 vs. 121.0 ± 57.2 mg/l), respectively, referring to Hoeller et al. [8]. Assuming an α error of 0.05 and a power of 0.95, calculated sample sizes were at least 75 patients in the non-infection group and 15 patients in the infection group, resulting in an actual power of 0.9532.

The distribution of data was assessed using the Shapiro–Wilk test. As the data were nonparametric, the Mann–Whitney test was performed. Nominal parameters were compared using χ^2^ test. Because blood samples could not be taken on a few days due to clinical circumstances (such as the need to conduct other necessary examinations), imputation was used to perform binary logistic regression in order to avoid losing any case. No patient was missing more than two of the seven blood samples. On average, 87.8% of all laboratory values were available (non-infection group: 87.4%, infection group: 90.4%). Significant correlations were selected to build a binary logistic regression model for determining infection predictors. Within the frame of an exploratory study, predictors were chosen through forward inclusion [19].

Statistical analysis was conducted with SPSS Statistics software version 29.0 (IBM SPSS Inc., Chicago, IL, USA), and GraphPad Prism 9.5.1 (GraphPad Software, San Diego, USA).

## Results

Seventeen of the 118 patients (14.4%) underwent revision surgery because of suspected SSI. In two of the patients who underwent revision (1.7%), the microbiologic examination result was negative for bacteria and there was no evidence of pus intraoperatively, so the patients were diagnosed with wound healing disorder. Their wounds healed after one revision, at which point, they were placed in the non-infection group. In the other 15 patients (12.7%), their microbiologic cultures showed evidence of bacterial growth, so they were placed in the infection group (see Table 1).

**Table 1:** Infection group patients Patients in the infection group are shown with their SSI [17] classifications and species

Patient	Day of revision	Classification of SSI	Species
1	8th	Deep	*Enterococcus faecalis, Staphylococcus capitis*
2	13th	Superficial	*Enterococcus faecalis, Staphylococcus epidermidis*
3	10th	Superficial	*Propionibacterium acnes*
4	14th	Superficial	*Proteus mirabilis, Escherichia coli*
5	13th	Superficial	*Staphylococcus aureus, Enterobacter cloacae complex*
6	7th	Deep	*Staphylococcus epidermidis*
7	7th	Deep	*Staphylococcus aureus, Enterobacter cloacae complex*
8	8th	Deep	*Staphylococcus aureus*
9	32nd	Deep	*Staphylococcus aureus*
10	12th	Superficial	*Proteus mirabilis, Staphylococcus epidermidis*
11	14th	Superficial	*Staphylococcus hominis*
12	43rd	Deep	*Staphylococcus aureus, Pseudomonas kuykendallii*
13	14th	Superficial	*Staphylococcus aureus*
14	13th	Superficial	*Staphylococcus epidermidis*
15	15th	Superficial	*Staphylococcus epidermidis*

With regard to the patient characteristics and comorbidities, the number of instrumented segments, the region of surgery and the presence of hypertension differed between the non-infection group and the infection group (see Table 2). Regarding the IMs, CRP_POD1,7_, PCT_POD7_, and IL-6_POD3,5,7_ were significantly higher in the infection group than in the non-infection group. IL-6_POD7_ showed an extremely high significance of P < 0.000001 (see Table 3).

**Table 2:** Baseline characteristics of patients who underwent dorsal spondylodesis in the non-infection and infection groups. Only the number of instrumented segments was significantly higher in the infection group than in the non-infection group. All other patient characteristics and comorbidities did not differ significantly between the groups

Variables	Patients, no. (%)	Patients, no. (%)	*P* value
	Non-Infection (*n* = 103)	Infection (*n* = 15)	
Patient-related factors			
Age, median (range), years	70 (56–76)	73 (62–78)	0.376^b^
Sex			0.931
Female	63 (61.2)	9 (60.0)	
Male	40 (38.8)	6 (40.0)	
ASA score			0.297
1	7 (6.8)	1 (6.7)	
2	65 (63.1)	6 (40)	
3	31 (29.1)	8 (53.3)	
BMI (kg/m^2^), median (range)	27.8 (23.4–31.5)	30.7 (25.3–36.3)	0.097^b^
< 18.5	0 (0)	0 (0)	0.155
18.5 to < 25	33 (32.0)	2 (13.3)	
25 to < 30	32 (31.1)	4 (26.7)	
≥ 30	38 (36.9)	9 (60)	
Comorbidities			
Smoking	21 (20.4)	2 (13.3)	0.519
Diabetes	14 (13.6)	2 (13.3)	0.978
Hypertonia	47 (45.6)	11 (73.3)	0.045
Thyroid disease	13 (12.6)	4 (26.7)	0.148
Osteoporosis	9 (8.7)	3 (20)	0.178
Kidney insuffiency	8 (7.8)	0 (0)	0.267
Coronary heart disease	14 (13.6)	3 (20)	0.509
Parkinson’s disease	6 (5.8)	0 (0)	0.337
Rheumatologic disease	9 (8.7)	1 (6.7)	0.788
Pulmonary disease	7 (6.8)	0 (0)	0.298
Coagulopathy	2 (1.9)	0 (0)	0.586
Hepatitis	2 (1.9)	1 (6.7)	0.277
Epilepsia	1 (1.0)	0 (0)	0.702
Neoplasia	8 (7.8)	2 (13.2)	0.470
Operation-related factors			
Indication for surgery			0.189
Degenerative disease	81 (78.6)	9 (60.0)	
Trauma	22 (21.4)	6 (40.0)	
Number of instrumented segments			0.037
1–3	84 (81.5)	9 (60)	
4–6	15 (14.6)	6 (40)	
> 6	4 (3.9)	0 (0)	
Region			0.015
Cervical	12 (11.7)	0 (0)	
Cervicothoracic	1 (1.0)	1 (6.$\overline{6 }$)	
Thoracic	9 (8.7)	4 (26.$\overline{6 }$)	
Thoracolumbar	9 (8.7)	0 (0)	
Lumbar	72 (69.9)	10 (66.$\overline{6 }$)	
Duration of surgery, median (range), min	184 (135–229)	192 (169–240)	0.247^b^

ASA: American society of anesthesiologists, BMI: Body mass index, NA: Not applicable

^a^χ^2^ Test, unless indicated otherwise. ^b^Mann-whitney *U* test

**Table 3:** Inflammatory markers (IMs). Of the five IMs, CRP_POD1,7_, PCT_POD7_, and IL-6_POD3,5,7_ were significantly higher in the infection group than in the non-infection group, with the highest significance of IL-6_POD7_ (*P* < *.*000001)

Infection marker		Non-infection group	Infection group	*P* value
		Median (range)	Median (range)	
WBC (10^3/µl)	Preoperatively	6.9 (5.9–8.3)	7.3 (6.4–8.3)	0.401
	POD 1	9.7 (8.3–11.8)	9.8 (7.7–13.0)	0.667
	POD 2	8.8 (7.1–10.5)	8.1 (7.3–12.5)	0.470
	POD 3	8.2 (6.3–10.0)	7.6 (6.1–12.2)	0.431
	POD 5	7.1 (5.3–9.0)	6.7 (5.8–8.3)	0.778
	POD 7	6.8 (4.7–9.3)	8.1 (6.3–9.6)	0.083
CRP (mg/l)	Preoperatively	2.8 (1.2–6.1)	3.8 (1.3–12.0)	0.178
	POD 1	33.0 (17.1–52.7)	56.5 (27.8–75.0)	0.049
	POD 2	82.3 (45.3–130.8)	124.0 (56.5–158.7)	0.185
	POD 3	96.3 (61.3–151.3)	110.2 (51.1–194.6)	0.426
	POD 5	67.4 (34.8–108.4)	82.2 (42.2–144.7)	0.279
	POD 7	37.79 (18.1–53.2)	69.5 (23.2–87.8)	0.008
PCT (µg/l)	Preoperatively	0.02 (0.02–0.03)	0.03 (0.02–0.04)	0.098
	POD 1	0.05 (0.03–0.11)	0.08 (0.05–0.11)	0.126
	POD 2	0.07 (0.04–0.1)	0.07 (0.05–0.18)	0.640
	POD 3	0.06 (0.03–0.09)	0.06 (0.04–0.21)	0.251
	POD 5	0.04 (0.02–0.07)	0.06 (0.03–0.22)	0.077
	POD 7	0.03 (0.02–0.05)	0.04 (0.03–0.15)	0.009
IL-6 (pg/ml)	Preoperatively	3.5 (2.1–5.9)	5.0 (3.1–11.0)	0.051
	POD 1	66.7 (26.6–100.7)	111.9 (58.9–134.0)	0.053
	POD 2	51.1 (32.4–86.3)	74.7 (33.5–159.7)	0.094
	POD 3	34.7 (21.6–66.0)	60.2 (30.8–138.7)	0.040
	POD 5	16.8 (11.4–26.1)	33.6 (14.6–47.8)	0.021
	POD 7	12.4 (6.8–18.8)	30.1 (22.3–33.8)	<0.001
TNF-α (pg/ml)	Preoperatively	7.4 (5.3–9.1)	7.9 (5.8–8.4)	0.621
	POD 1	8.3 (6.4–9.9)	8.3 (6.4–10.0)	0.890
	POD 2	9.7 (7.3–12.0)	9.2 (7.6–15.8)	0.726
	POD 3	10.4 (7.8–13.1)	11.8 (8.1–17.8)	0.214
	POD 5	9.4 (6.6–11.8)	10.2 (6.5–15.2)	0.248
	POD 7	9.11 (6.8–11.4)	9.6 (7.0–11.1)	0.843

*WBC* White blood cell, *POD* postoperative day, *CRP* C-reactive protein, *PCT* Procalcitonin, *IL-6* Interleukin-6, *TNF-α* Tumor necrosis factor α

**Fig.1:**
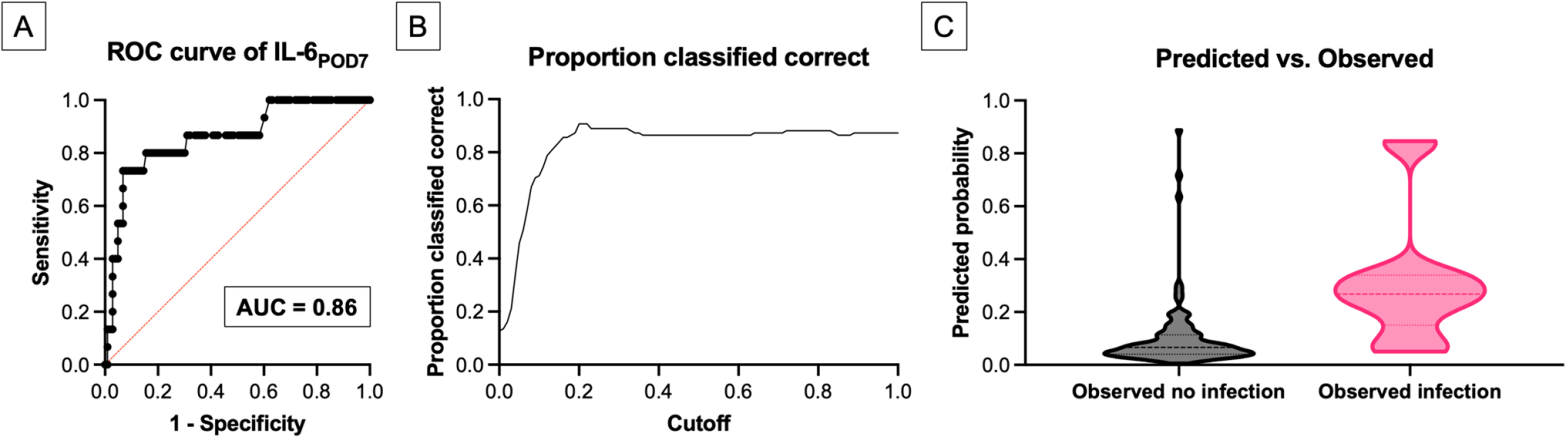
Results of the binary regression analysis for IL-6. **A** The receiver operating characteristic curve of IL-6_POD7_ with an AUC of 0.86. **B** The proportion of correct classifications, with the best cutoff at 0.2, corresponding to an IL-6_POD7_ level of 26.0 pg/mL. **C** The probability of predicting an SSI using IL-6_POD7_. *IL-6* Interleukin-6, *AUC* Area under the curve

Binary logistic regression analysis for SSI detection was first performed using all significantly different baseline characteristics and IMs between the two groups (hypertonia, number of instrumented segments, region of surgery; CRP_POD1,7_, PCT_POD7_, and IL-6_POD3,5,7_). Significant regression coefficients were observed only for the three IL-6 values on PODs 3, 5, and 7 (IL-6_POD3_: odds ratio [OR] = 1.03; 95% CI 1.01–1.05; P = 0.023; IL-6_POD5_: OR = 0.94; 95% CI 0.89–0.99; P = 0.021; IL-6_POD7_: OR = 1.13; 95% CI 1.05–1.23; P = 0.001). The receiver operating characteristic (ROC) showed that the model including all significant parameters (hypertonia, number of instrumented segments, region of surgery; CRP_POD1,7_, PCT_POD7_, and IL-6_POD3,5,7_) had an area under the curve (AUC) of 0.88 (95% CI 0.79–0.98; P < 0.001) and a Nagelkerke’s R^2^ of 0.46. The binary logistic regression for IL-6_POD3,5,7_ had an AUC of 0.86 (95% CI 0.74–0.97; P < 0.001) and a Nagelkerke’s R^2^ of 0.38. Additional models were systematically tested. Initially, each IM from each day was tested individually, revealing the main effect of IL-6_POD7_ on SSI detection. The binary logistic regression analysis using only IL-6_POD7_ showed a highly significant regression coefficient (OR = 1.10; 95% CI 1.05–1.16; P = 0.0002), an AUC of 0.86 (95% CI 0.75–0.97; P < 0.001), a Nagelkerke’s R^2^ of 0.29, and a strong effect with Cohen’s f^2^ of 0.40 (Fig. 1A). The second-best IMs for SSI detection were CRP_POD7_ (AUC, 0.71; 95% CI 0.56–0.86; P = 0.009; Nagelkerke’s R^2^: 0.13) and PCT_POD7_ (AUC, 0.70; 95% CI 0.56–0.84; P = 0.011; Nagelkerke’s R^2^: 0.18), while all other IMs showed even less predictive potential with respect to the AUC. The optimal cutoff for the model using only IL-6_POD7_ was set at 0.2, corresponding to an IL-6 level of 26.0 pg/mL (Fig. 1B). This allowed for SSI detection with a sensitivity of 0.73, a specificity of 0.93, a positive predictive value (PPV) of 0.61, and a negative predictive value (NPV) of 0.96 (Fig. 1C).

**Fig.2:**
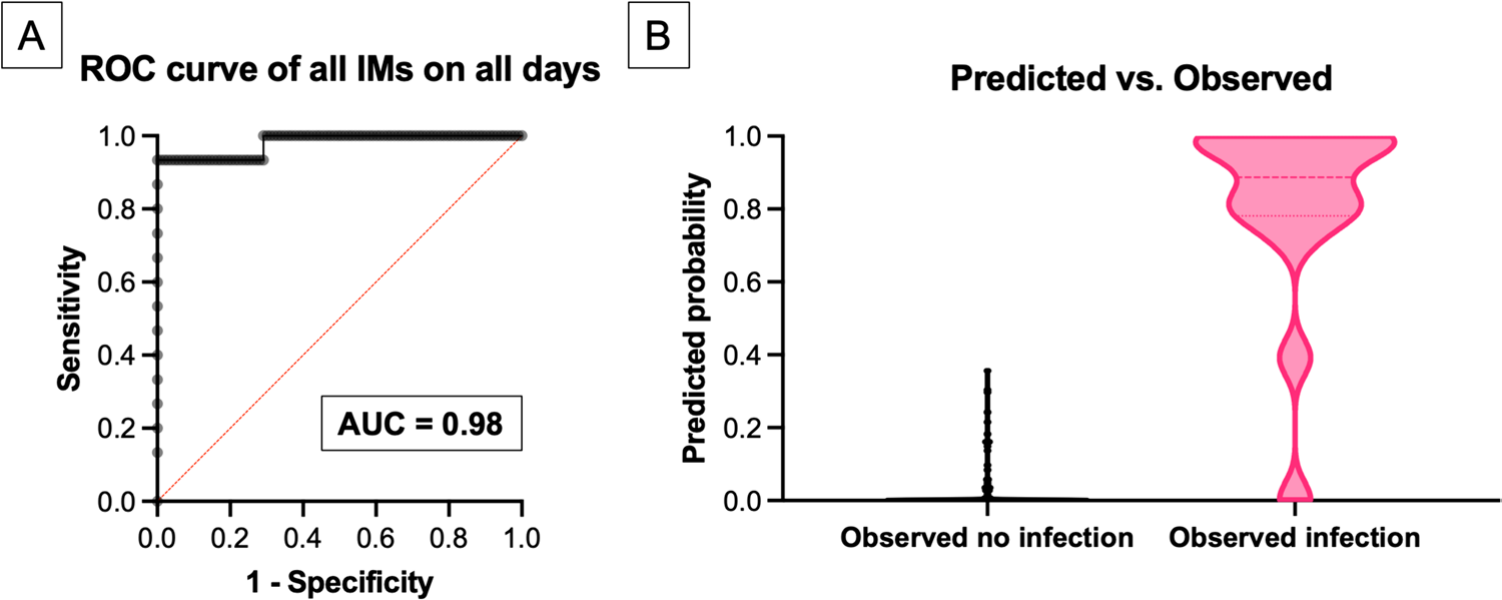
Results of the binary regression analysis of all IMs for all days. **A** The receiver operating characteristic curve of all the IMs on all days, with an AUC of 0.98. **B** Probability of predicting an SSI using all the IMs on all days. *IM* Inflammatory marker, *AUC* Area under the curve, *SSI* Surgical site infection

Further models were tested as follows: Starting with IL-6_POD7_, the other IMs—PCT, CRP, TNF-α, and WBC count from POD 7—were sequentially added to the model. Subsequently, the IMs from PODs 5, 3, 2, and 1 were added sequentially to the models in the aforementioned order, revealing again the main effect of IL-6_POD7_ on SSI detection. In general, as more IMs were used in the binary logistic regression, the AUC increased, ultimately resulting in an AUC of 0.98, a Nagelkerke’s R^2^ of 0.80, a sensitivity of 0.87, a specificity of 1.0, a PPV of 1.0, and an NPV of 0.98 when all IMs from all days were used (Fig. 2).

## Discussion

To date, the indication for revision surgery for SSI is based on wound presentation, supported by IMs, particularly, CRP, and, in some cases, by imaging modalities. CRP was shown to have specific post-operative dynamics in the case of SSI as a prolonged plateau and a later second peak at about POD 10 that might indicate revision surgery [8]. Nevertheless, there is no reference test using CRP that allows for safe and early SSI diagnosis.

Our data show that the critical IM for early SSI detection is IL-6. Furthermore, compared to CRP, WBC count, PCT, and TNF-α, IL-6 appears to be the only IM that can indicate SSI up to POD 7. This is consistent with observations that IL-6 is immediately synthesized in response to infection, activating an acute immune response and inducing CRP production by hepatocytes [9, 20].

Meisner [21] showed that after surgical trauma, IL-6 and TNF-α are the first IMs to increase, followed by PCT and finally, CRP. Because both the non-infection and infection groups in our study had suffered surgical trauma, there was no initial difference in IL-6 in our study. Only after the onset of early infection was there was a measurable difference in the immune response. We propose that analogous to the sequence of increases in IMs after surgical trauma, the acute immune response in infection is initiated by IL-6 [9, 20]. The value of IL-6 in SSI diagnosis was also observed by Lenski et al. [22], who showed an AUC of 0.95 for IL-6 in SSI prediction. Notably, however, in their study, only 9 patients were infected out of 89, and 8 of those 9 patients had late deep infections with discitis or epidural abscess and underwent revision surgery on average at POD 49. In line with this study of Lenski et al. [22] was that of Berbari et al. [23], whose meta-analysis showed that IL-6 generally seemed to have had the best diagnostic accuracy for prosthetic joint infection compared to CRP and WBC count but who clearly stated that early and late infections were not differentiated.

Rettig et al. [10] found that high levels of IL-6 were associated with postoperative complications after major abdominal surgery that included not only SSIs but also pneumonia, urinary tract infection, and others. In their analysis, the difference in IL-6 between the group with complications and the group without complications was observed at POD 1, which might also have been associated with the surgical trauma [21]. In addition, Rettig et al. [10] observed differences in CRP_POD3,7_ and in TNF-α_POD7_, while WBC count did not differ between the groups. Rettig et al. [10] did not report a multiple comparison correction that might have led to more results with lower significance levels. Although the use of multiple comparison correction is controversial [24], when applied it to our results retrospectively (Holm-Šídák method), it confirmed the highly significant effect of IL-6_POD7_ (P < 0.000001) as the only remaining significantly different IM.

Two patients from the infection group underwent late revision surgery (Patient 9 on POD 32 and Patient 12 on POD 43). However, at that time, the primary IMs (i.e., CRP and WBC count) were not significantly elevated, with only one peak after surgery until their initial discharge (Patient 12: highest postoperative CRP at 95.5 mg/l on POD 3, and Patient 12: highest postoperative CRP at 38.2 mg/l on POD 4). In both patients, IL-6_POD7_ was below the cutoff value. Thus, both patients would have been predicted false-negatively using IL-6_POD7_. This might indicate different IL-6 dynamics between very early and early SSIs. Both patients were placed in the infection group because a clearly visible SSI (wound secretion, wound dehiscence) had developed within the usual range for early SSI of 30 days [17]. However, revision surgery for patient 9 was prolonged by about 30 h due to capacity problems. Patient 12 had initially been readmitted to another hospital, where unfortunately, conservative treatment was started with the administration of antibiotics.

Both PCT and TNF-α have value in the diagnosis and monitoring of sepsis [25]. Nie et al. [26] found better predictive values for PCT than for CRP in patients with acute traumatic spinal cord injury. Aouifi et al. [15] showed that PCT may be more reliable than CRP as an IM for SSI diagnosis after cardiac surgery. Although we observed a significant difference between the two groups in PCT_POD7_, we could not show a relevant predictive value for SSI prediction. Little is known about the predictive value of TNF-α for SSIs. We found no difference between the two groups in TNF-α, in line with the finding of Bottner et al. [27] that TNF-α was not relevant in prosthetic joint infections.

By including all IMs from all PODs, we were able to increase the AUC to 0.98, which resulted in an almost certain SSI diagnosis. Notably, though, this approach does not seem to be a realistic option in practice due to the costs involved.

Regarding the patient characteristics and comorbidities, the number of instrumented segments, the region of surgery, and the presence of hypertension significantly differed between the groups, while current risk factors, such as diabetes mellitus, obesity, use of steroids, drainage time, and operative time, were insignificant [28], possibly because the cohort was too small.

Our observed SSI rate of 12.7% is slightly higher than the range reported in the current literature, which indicates the risk of SSIs after dorsal spondylodesis to be approximately 0.7%-11.9% [29]. Given that our study was conducted at a maximum care spine center (Level 1) of the German Spine Society (DWG®) within a university medical center, it is plausible that our patient cohort exhibited higher levels of illness, more challenging surgeries, and age compared to those seen in non-maximum care centers. This is evident from the distribution of ASA and BMI scores, where a greater proportion of patients in both groups exhibited higher ASA classifications and BMIs above 25 kg/m^2^.

In conclusion, in this study, compared to CRP, PCT, and TNF-α, IL-6 had the highest value for early SSI diagnosis. Based on the results in this cohort, an SSI would be diagnosed if the IL-6_POD7_ value is 26.0 pg/mL or higher. Because most cases of superficial wound infections can be managed without surgery by administering antibiotics[30], our results indicate a false-positive rate of 6.8% and correct antibiotic administration in 61% of the patients starting on POD 7. Early initiation of antibiotics could significantly reduce the number of revisions required. Nevertheless, in light of the present results, the future role of low-cost CRP may need to be reconsidered.

This study had limitations. First, the IMs were measured before the surgery and on PODs 1, 2, 3, 5, and 7. The measurement of the IMs on all days might have influenced the results. Second, this was a single-center prospective diagnostic study with a relatively small cohort of patients. Although the sample size was calculated and the cohort was large enough, a multicenter study with more patients would have strengthened the results. Third, although no differences between non-infection and infection groups were observed in preoperative values of age, CRP, and IL-6, all these parameters were higher in the infection groups compared to the non-infection group, particularly when considering the preoperative p value of IL-6 (p = 0.051), indicating at least a trend between groups. While we endeavor to account for all comorbidities to minimize bias in the development of an SSI, the use of corticosteroids or history of splenectomy was not accounted for, and nutrition was assessed solely by BMI, without specific malnutrition scores. Thus, an impaired immune status due to immunosuppressive status, malnutrition, or aging may have contributed to perioperative inflammation. Fourth, although statistical analysis did not reveal differences between groups regarding the underlying indication of surgery, the mix of both degenerative and traumatic pathologies could have potentially influenced the outcomes.

## Data Availability

No datasets were generated or analysed during the current study.

## Abbreviations

AUC: Area under the curve
CRP: C-reactive protein
IL-6: Interleukin-6
IM: Inflammatory marker
NPV: Negative predictive value
PCT: Procalcitonin
POD: Postoperative day
PPV: Positive predictive value
ROC: Receiver operating characteristic
SSI: Surgical site infection
TNF-α: Tumor necrosis factor α
WBC: White blood cell
