# Correction: Interleukin-6 as a critical inflammatory marker for early diagnosis of surgical site infection after spine surgery

**DOI:** 10.1007/s15010-024-02319-5

**Published:** 2024-06-13

**Authors:** Paul Jonathan Roch, Carolin Ecker, Katharina Jäckle, Marc-Pascal Meier, Maximilian Reinhold, Friederike Sophie Klockner, Wolfgang Lehmann, Lukas Weiser

**Affiliations:** 1 Department of Trauma Surgery, Orthopaedics and Plastic Surgery, University of Göttingen, Robert-Koch-Str. 40, 37075 Göttingen, Germany; 2 Department of Surgery, University Medical Center Mannheim, Theodor-Kutzer-Ufer 1-3, 68167 Mannheim, Germany

**Infection**

10.1007/s15010-024-02271-4

In this article the affiliation details for all Authors were incorrectly given as

Paul Jonathan Roch^1,2^ · Carolin Ecker^1,2^ · Katharina Jäckle^1,2^ · Marc-Pascal Meier^1,2^ · Maximilian Reinhold^1,2^ · Friederike Sophie Klockner^1,2^ · Wolfgang Lehmann^1,2^ · Lukas Weiser^1,2^

but should have been

Paul Jonathan Roch^1^ · Carolin Ecker^2^ · Katharina Jäckle^1^ · Marc-Pascal Meier^1^ · Maximilian Reinhold^1^ · Friederike Sophie Klockner^1^ · Wolfgang Lehmann^1^ · Lukas Weiser^1^

The Original Article has been corrected.

